# Development and internal validation of a nomogram for predicting survival of nonoperative EGFR-positive locally advanced elderly esophageal cancers

**DOI:** 10.3389/fonc.2023.1097907

**Published:** 2023-05-12

**Authors:** Jiayang Wang, Jin Peng, Honglei Luo, Yaqi Song

**Affiliations:** Department of Radiation Oncology, The Affiliated Huaian No.1 People’s Hospital of Nanjing Medical University, Huaian, Jiangsu, China

**Keywords:** esophageal cancer, unresectable, nomogram, icotinib, stage, ECOG score

## Abstract

**Purpose:**

This study aims to develop and validate a prediction model for non-operative, epidermal growth factor receptor (EGFR)-positive, locally advanced elderly esophageal cancer (LAEEC).

**Methods:**

A total of 80 EGFR-positive LAEEC patients were included in the study. All patients underwent radiotherapy, while 41 cases received icotinib concurrent systemic therapy. A nomogram was established using univariable and multivariable Cox analyses. The model’s efficacy was assessed through area under curve (AUC) values, receiver operating characteristic (ROC) curves at different time points, time-dependent AUC (tAUC), calibration curves, and clinical decision curves. Bootstrap resampling and out-of-bag (OOB) cross-validation methods were employed to verify the model’s robustness. Subgroup survival analysis was also conducted.

**Results:**

Univariable and multivariable Cox analyses revealed that icotinib, stage, and ECOG score were independent prognostic factors for LAEEC patients. The AUCs of model-based prediction scoring (PS) for 1-, 2-, and 3-year overall survival (OS) were 0.852, 0.827, and 0.792, respectively. Calibration curves demonstrated that the predicted mortality was consistent with the actual mortality. The time-dependent AUC of the model exceeded 0.75, and the internal cross-validation calibration curves showed good agreement between predicted and actual mortality. Clinical decision curves indicated that the model had a substantial net clinical benefit within a threshold probability range of 0.2 to 0.8. Model-based risk stratification analysis demonstrated the model’s excellent ability to distinguish survival risk. Further subgroup analyses showed that icotinib significantly improved survival in patients with stage III and ECOG score of 1 (HR 0.122, P<0.001).

**Conclusions:**

Our nomogram model effectively predicts the overall survival of LAEEC patients, and the benefits of icotinib were found in the clinical stage III population with good ECOG scores.

## 1 Introduction

Currently, concurrent chemoradiotherapy (CCRT) is the preferred treatment for unresectable locally advanced elderly esophageal carcinoma (LAEEC) (1–3). However, elderly patients often struggle to tolerate CCRT due to their poor performance status. Studies have demonstrated that only about one-third of elderly patients can complete concurrent chemoradiotherapy, which fails to improve their survival compared to radiotherapy alone, resulting in a median overall survival (OS) of less than 2 years (4–8). Thus, there is a pressing need to explore new therapeutic strategies for elderly patients with unresectable esophageal cancer.

Targeted therapy has emerged as a low-toxicity, high-efficiency antitumor systemic therapy (9–12). Epidermal growth factor receptor (EGFR) tyrosine kinase inhibitors (TKIs) represent the most common targeted drugs and play a crucial role in treating non-small cell lung cancer with EGFR gene mutations (10, 13–15). Research has shown that several EGFR-TKIs, including gefitinib and icotinib, exhibit a favorable safety profile in elderly patients, with significantly fewer adverse effects than cytotoxic drugs (16–20). Among these, icotinib has been reported to have lower adverse effects than gefitinib and better suitability for elderly patients or those with poor performance scores (21). Concurrently, EGFR overexpression has been observed in approximately 30-70% of esophageal cancers (22), and EGFR-TKIs have been demonstrated to disrupt cell proliferation and enhance the radiosensitivity of cancer cells (23, 24). This suggests that EGFR-TKIs could be a promising treatment option for EGFR-positive esophageal cancer.

Previous studies have shown that many EGFR TKIs, including erlotinib (23, 25, 26), gefitinib (27), cetuximab (15, 16) and icotinib (28) are effective and safe for patients with esophageal squamous cell carcinoma (ESCC). However, most of these studies focused on the combination of EGFR-TKIs with chemotherapy in metastatic esophageal cancer patients, with limited research on LAEEC. Therefore, we designed this study to investigate the efficacy of icotinib and establish a prediction model of icotinib for EGFR-positive LAEEC population, aiming to provide valuable insights into potential treatment strategies for this population.

## 2 Methods

### 2.1 Patients

**Figure 1:**
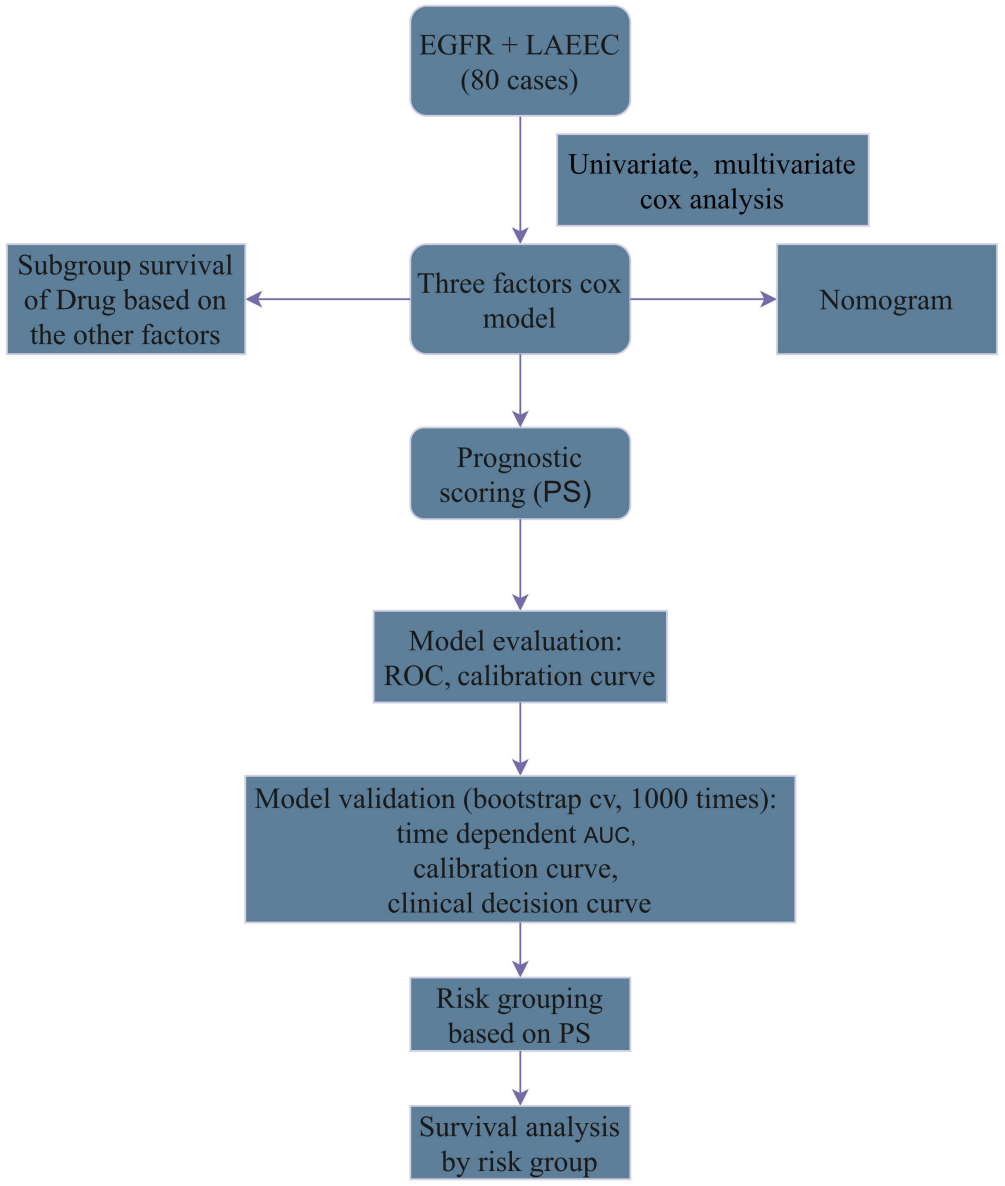
Study Flowchart: Patient Selection and Analysis.

A total of 80 LAEEC patients who received primary treatment at our hospital between 2014 and 2018 were enrolled in this study. All patients underwent radical radiation therapy with a dose of 60Gy/30F (2), and 41 of them received icotinib. Inclusion criteria were as follows: 1) pathologically diagnosed esophageal squamous carcinoma with immunohistochemical (IHC) confirmed overexpression of EGFR, 2) age >= 70 years, 3) limited stage (stage II-III, AJCC 7th) (29), 4) intolerance to surgery or refusal of surgery, and 5) complete treatment and have complete clinical pathology data. The flow chart is displayed in **Figure 1**.

This study was approved by the review committee of the Affiliated Huaian No.1 People’s Hospital of Nanjing Medical University. Informed consent was waived as it was a retrospective study.

### 2.2 Follow-up

Overall survival was defined as the time from initial treatment to the end of follow-up or death. All patients were followed up until 2022-08-01, with a median follow-up duration of 1.9 years.

### 2.3 Treatment procedures

All patients received radical radiation therapy (RT) using 6Mv X-rays through 3D conformal radiation therapy or intensity-modulated radiation therapy. Gross tumor volume (GTV) comprised the primary tumor and any metastatic regional lymph nodes. Clinical target volume (CTV) included total tumor volume, 3-5 cm longitudinal extensions along the esophagus, 0.5-1 cm horizontally extension, and regional lymph nodes. PGTV and PTV were defined as 0.5-1 cm outward expansion of GTV and CTV, receiving a dose of 60 Gy/30F/6W and 40-50Gy/20-25F/4-5W, respectively. Icotinib was administered at 125 mg, orally, three times daily, concurrently with RT.

### 2.4 Statistical analysis

Statistical analyses were conducted using R 4.2.1 (30) (R Foundation for Statistical Computing, Vienna, Austria). Age and tumor length were grouped by optimal cut-off point calculated by the “survminer” package (31). In this study, a total of 60 patients were confirmed dead. As OS was the positive event, according to the principle of 10 events per variable (10 EPV), the number of variables included in the final model should not exceed six. Categorical variables were expressed as numbers (percentages), and continuous data were expressed as mean ± standard deviation (SD). Differences between groups were compared using the chi-square test or Fisher’s exact test for categorical variables and the ANOVA test for continuous variables. Univariable and multivariable Cox analyses were employed to select the prognostic factors and develop a predictive model. Bidirectional forward and backward stepwise regression was utilized to optimize the model. A nomogram and a prognostic score (PS) were established based on the optimized model. The time-dependent ROC curve, calibration curve and decision curve were plotted by the “timeROC”, “riskRegression”, and “dcurves” packages (32–34) to evaluate the model’s performance. The “riskRegression” package (33) was employed to calculate the 1000-time average AUC, plot the time-dependent AUC curve and the calibration curve to verify the model’s robustness *via* bootstrap resampling and the internal cross-validation method outside the bag (OOB). The “survminer” package (31) was used to select the cut-off values, divide the patients into high-risk and low-risk groups, and compare the survival differences between the two groups with log-rank test. Finally, The survival benefit of icotinib in various subgroup populations was analyzed with log-rank test to identify the beneficiary population of icotinib. Univariable analysis was considered statistically significant at p < 0.1, and other analyses were considered statistically significant at p < 0.05.

## 3 Results

### 3.1 Patient characteristics

A total of 80 patients were included in this study, and their baseline characteristics are presented in **Table 1**. Nine factors were considered, including icotinib treatment, gender, age (≤78 years vs. >78 years), tumor length (<5 cm vs. ≥5 cm), tumor location (upper vs. middle vs. lower), clinical stage (II vs. III, AJCC 7th), EGFR expression ((+, Low) vs. (++~+++, High)) (**Figure S1**)), weight loss of more than 5% within 6 months, and ECOG score. Patients were divided into two groups based on icotinib treatment. No significant differences in clinical characteristics were observed between the two groups, except for EGFR expression.

**Table 1:** Baseline and clinical characteristics of the patients grouped by drug.

Characteristics	Overall, N = 80^1^	Without icotinib, N = 39^1^	With icotinib, N = 41^1^	p-value^2^
**Sex**				0.244
Female	46 (57%)	25 (64%)	21 (51%)	
Male	34 (42%)	14 (36%)	20 (49%)	
**Age**				0.784
Old	32 (40%)	15 (38%)	17 (41%)	
Young	48 (60%)	24 (62%)	24 (59%)	
**Location**				0.281
Lower	21 (26%)	13 (33%)	8 (20%)	
Middle	35 (44%)	14 (36%)	21 (51%)	
Upper	24 (30%)	12 (31%)	12 (29%)	
**Length**				0.352
≥5 cm	45 (56%)	24 (62%)	21 (51%)	
< 5 cm	35 (44%)	15 (38%)	20 (49%)	
**Stage**				0.379
II	43 (54%)	19 (49%)	24 (59%)	
III	37 (46%)	20 (51%)	17 (41%)	
EGFR				0.010
Low	24 (30%)	17 (44%)	7 (17%)	
High	56 (70%)	22 (56%)	34 (83%)	
Weight				1.000
No	10 (12%)	5 (13%)	5 (12%)	
Yes	70 (88%)	34 (87%)	36 (88%)	
ECOG				0.180
1	53 (66%)	23 (59%)	30 (73%)	
2	27 (34%)	16 (41%)	11 (27%)	

^1^n (%).

^2^Pearson’s Chi-square test; Fisher’s exact test.

### 3.2 Univariable and multivariable analysis

Univariable and multivariable analysis results are displayed in **Table 2**. Univariable cox analysis identified icotinib treatment (p < 0.001), clinical stage (p = 0.031), EGFR expression (p = 0.038), and ECOG score (p = 0.006) as potential factors affecting overall survival (OS). A multivariable Cox analysis was performed using these four factors (model 1) and optimized with stepwise regression (model 2). The finally results demonstrated that clinical stage (III vs II, HR 1.72, 95% CI 1.02~2.90), icotinib treatment (Y vs N, HR 0.29, 95% CI 0.16~0.52), and ECOG score (2 vs 1, HR 2.02, 95% CI 1.18~3.46) were independent prognostic factors for OS.

**Table 2:** Univariable and multivariable cox analysis for OS of LAEEC patients.

	Uni-variable				Multi-variable1(model1)			Multi-variable2(model2)		
Characteristics	N	HR^1^	95% CI^1^	p-value	HR^1^	95% CI^1^	p-value	HR^1^	95% CI^1^	p-value
Icotinib	80									
No		—	—		—	—		—	—	
Yes		0.30	0.17, 0.53	<0.001	0.31	0.17, 0.60	<0.001	0.29	0.16, 0.52	<0.001
Sex	80									
female		—	—							
male		0.89	0.53, 1.51	0.667						
Age	80									
Old		—	—							
Young		1.50	0.89, 2.54	0.129						
Location	80									
Lower		—	—							
Middle		0.97	0.52, 1.82	0.928						
Upper		0.68	0.33, 1.37	0.276						
Length	80									
≥5 cm		—	—							
< 5 cm		0.75	0.44, 1.25	0.265						
Stage	80									
II		—	—		—	—		—	—	
III		1.76	1.05, 2.93	0.031	1.80	1.06, 3.07	0.030	1.72	1.02, 2.90	0.041
EGFR	80									
Low		—	—		—	—				
High		0.55	0.31, 0.97	0.038	0.76	0.40, 1.43	0.391			
Weight	80									
No		—	—							
Yes		1.88	0.85, 4.16	0.119						
ECOG	80	2.09	1.23, 3.56	0.006						
1					—	—		—	—	
2					1.96	1.14, 3.38	0.015	2.02	1.18, 3.46	0.011

^1^HR, Hazard Ratio; CI, Confidence Interval.

### 3.3 Development and validation of the nomogram

**Figure 2:**
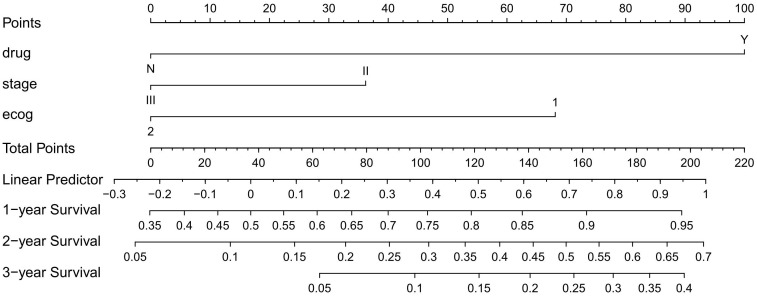
Nomogram for Predicting Overall Survival in LAEEC Patients.

**Figure 3:**
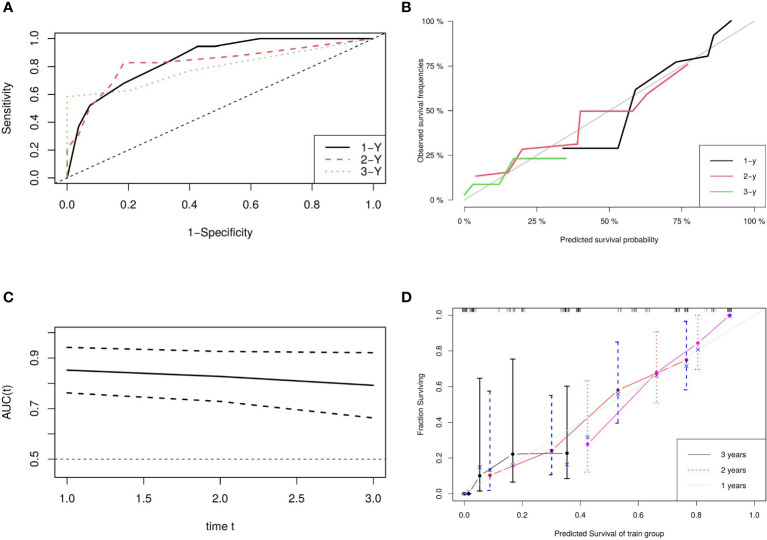
Model Evaluation and Validation: **(A)** ROC Plots for Predictive Score, **(B)** Calibration Curves for Predictive Score, **(C)** Time-Dependent AUC, and **(D)** Bootstrap Cross-Validation Calibration Curve (1,000 Resamples).

**Figure 4:**
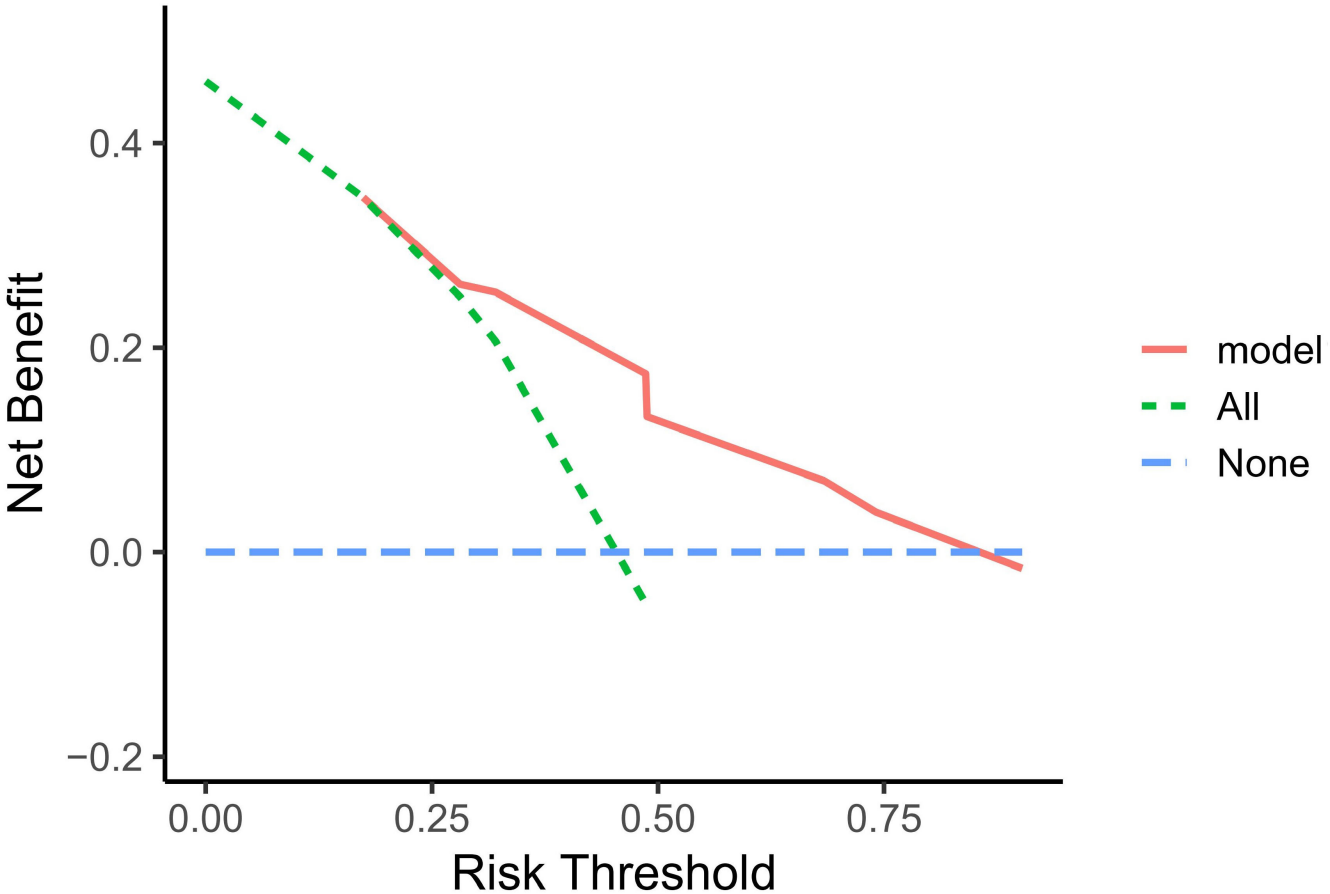
Decision Curve Analysis of the LAEEC Patient Prediction Model.

A nomogram based on model 2 was created to predict OS in LAEEC patients (**Figure 2**), and a prognostic score (PS) was established using the following formula: 0.545 * stage (III) - 1.252 * drug (Y) + 0.703 * ecog (2). The time-dependent ROC curves (**Figure 3A**) revealed the AUCs of 0.852 (95% CI 0.762 - 0.942), 0.827 (95% CI 0.728 – 0.926), and 0.792 (95% CI 0.663 – 0.921) for 1-year, 2-year, and 3-year OS, respectively. Calibration curve plots (**Figure 3B**) indicated that the predicted survival probability closely matched the actual survival probabilities at 1, 2, and 3 years, suggesting good predictive accuracy. Time dependent AUC (**Figure 3C**) and calibration curves (**Figure 3D**) were obtained using the bootstrap internal cross-validation method with the “riskRegression” package. The AUC exceeded 0.75 throughout the study period, and the calibration curve closely aligned with the standard line, confirming the model’s robust predictive performance and stability. The decision curve (**Figure 4**) demonstrated that the model provided a better net clinical benefit than reference curves for threshold probabilities from 0.2 to 0.8.

### 3.4 Risk stratification based on the nomogram

**Figure 5:**
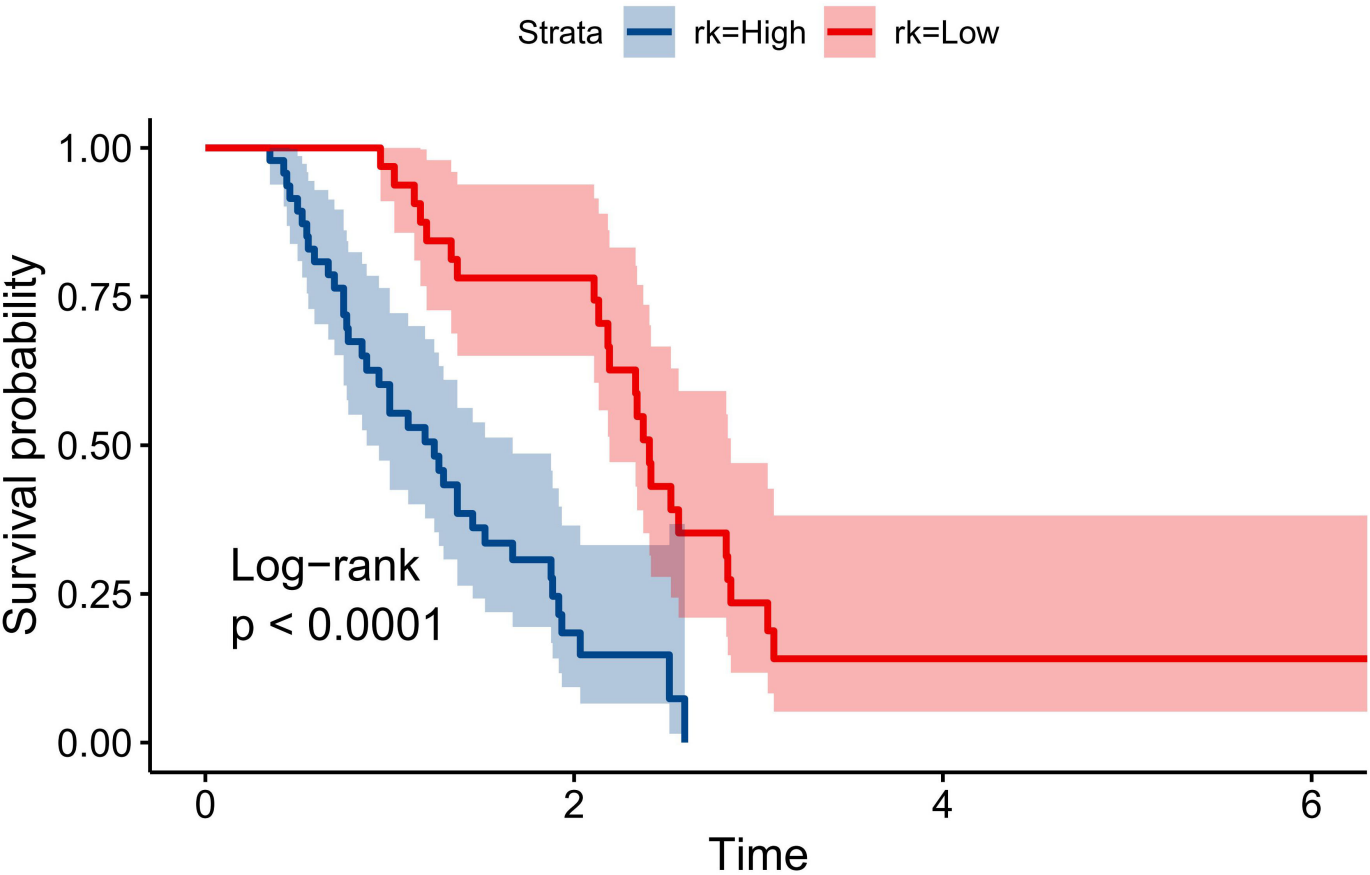
Comparison of Overall Survival Curves for Low-Risk and High-Risk LAEEC Patients.

Patients were divided into high- and low-risk groups based on PS, using a cut-off point of -0.549 by the “survminer” package. The risk of death was significantly lower in the low-risk group compared to the high-risk group (HR 0.24, 95% CI 0.13-0.45, P<0.001) (**Figure 5**).

### 3.5 Subgroup survival analyses

**Figure 6:**
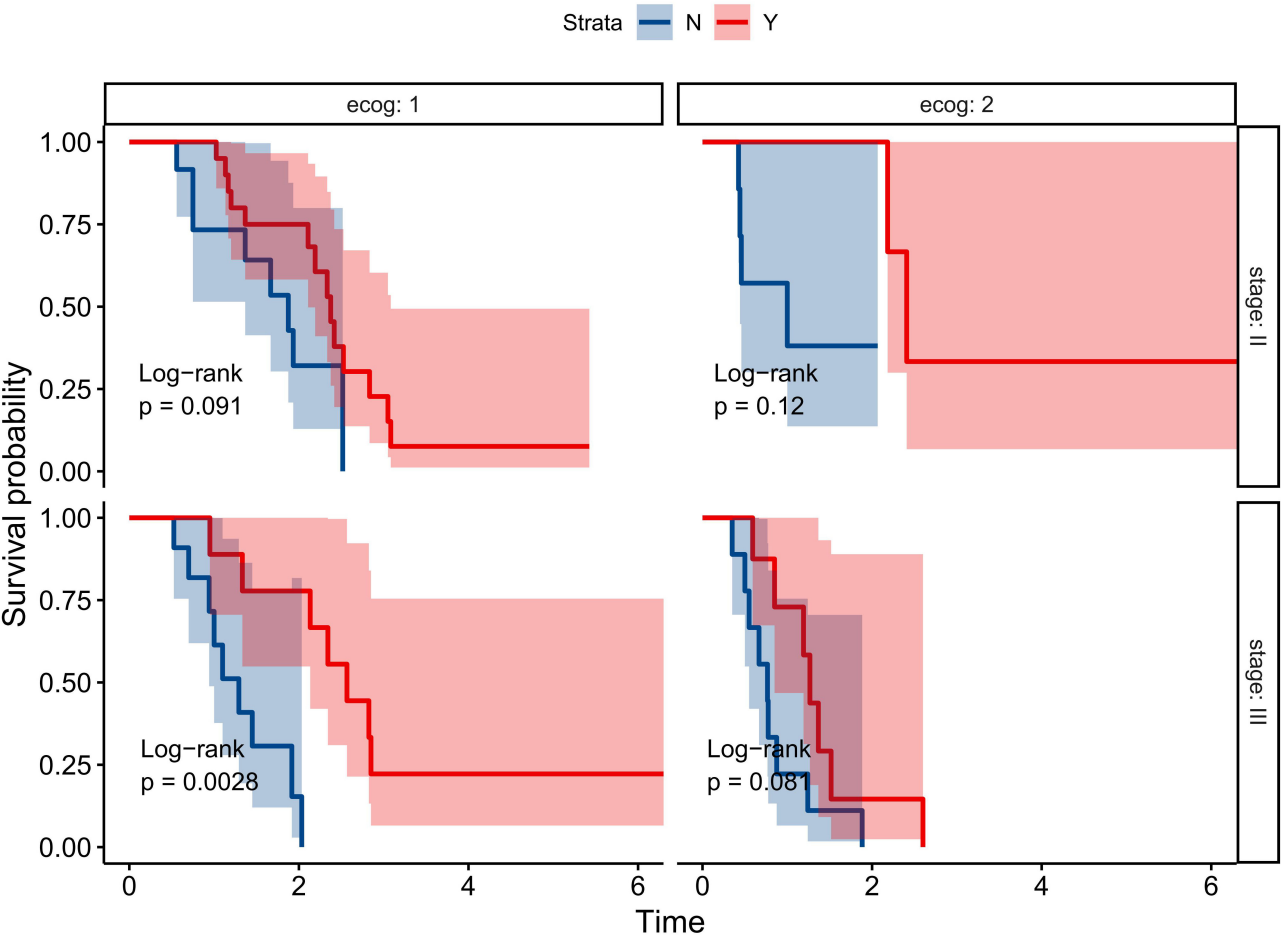
Subgroup Survival Analysis of Icotinib Treatment Stratified by Stage and ECOG Score.

Subgroup analysis, stratified by prognostic factors such as clinical stage and ECOG score, demonstrated that icotinib significantly improved OS only in the clinical stage III group with a good ECOG score (HR 0.122, 95% CI 0.0248 ~ 0.597, P<0.001). However, no significant improvement in OS was observed in other subgroups (**Figure 6**).

## 4 Discussion

Icotinib, an oral EGFR-TKI, modulates cell survival and proliferation by inhibiting the phosphorylation of tyrosine kinase in downstream signaling proteins, obstructing the activation of intracellular signaling pathways, and ultimately regulating the transcription of target genes (35). With EGFR being widely expressed in various solid cancers, icotinib is essential in treating numerous solid malignancies (15, 36–40). Both the EVIDENCE (38) and CONVINCE (41) studies demonstrated that icotinib considerably reduced DFS and serious adverse effects compared to chemotherapy for patients with EGFR-mutated lung adenocarcinoma. The ICAPE study (36) confirmed icotinib’s safety and efficacy in treating EGFR mutant lung adenocarcinoma, regardless of the EGFR mutation type. Zhang et al. (42) found that icotinib enhanced the radiation sensitivity of lung cancer cells by inhibiting the activation of MAPK/ERK/AKT pathway. Zhao et al. (37) indicated that icotinib, combined with concurrent radiotherapy, was safe and effective in the treatment of locally advanced squamous cervical cancer. Wang et al. (28) reported the favorable efficacy and safety of icotinib as a standalone treatment for EGFR-mutated esophageal cancer, suggesting that icotinib could be a viable treatment for elderly patients (43). Our previous study (44) also showed that icotinib combined with radiation therapy was safe and effective for older patients with esophageal cancer, particularly those with EGFR overexpression. Consequently, we designed this study to analyze the impact of icotinib combined with radiotherapy on the prognosis of EGFR-positive LAEEC patients, and to establish and validate a predictive model for clinical decision-making.

In this study, we developed a three-factor proportional risk model, including stage, ECOG, and drug, using cox analysis. The results revealed that the HR of OS in stage III patients were 72% higher than in stage II (HR 1.72, 95% CI 1.02~2.90), and the HR of OS in patients with pool performance status (ECOG 2) was higher than in those with good performance status (ECOG 1) (HR 2.02, 95% CI 1.18~3.46). Icotinib reduced the OS risk by 71% (HR 0.29, 95% CI 0.16~0.52). We then established a predictive nomogram based on the model. The AUCs of the model were 0.852, 0.827, and 0.792 at 1, 2, and 3 years, respectively. Calibration curves demonstrated high agreement between predicted and actual probabilities, and the decision curve displayed a net benefit in the range of 20%-80%. Bootstrap cross-validation by 1000-time resampling indicated that the time-dependent AUC consistently exceeded 0.75, and the calibration curve was close to the diagonal, suggesting that the model had good stability. The survival analysis based on risk stratification by PS showed that the risk of death in the low-risk group was significantly lower than that in the high-risk group (HR 0.243, P<0.001), indicating that the model could effectively identify the death risk of EGFR-position LAEEC patients. Lastly, subgroup analysis revealed that icotinib could significantly reduce the death risk in the clinical stage III, ECOG 1 population. These findings would aid clinicians in assessing the prognosis of elderly patients with EGFR positive and provide a recommendation for icotinib use.

This study, however, had some limitations. First, the cases were collected from 2014 to 2018, during which genetic sequencing technology was not widely available. As a result, EGFR expression in our study was tested using IHC, and the expression of the EGFR resistance gene was not considered. This might also explain why EGFR expression did not emerge as an independent prognostic factor. In subsequent studies, We will introduce the expression of EGFR genes and EGFR resistance genes to optimize our model. Second, this study was a single-institution retrospective study with a small sample size. Although we used bootstrap cross-validation and internal resampling method, selection bias was still inevitable. Therefore, more rigorous prospective studies were needed to validate our conclusions. Overall, our findings contribute to the current understanding of the role of icotinib in treating EGFR-positive LAEEC patients and offer valuable insights for clinical decision-making.

## 5 Conclusions

In conclusion, stage, ECOG score and icotinib have been identified as independent prognostic factors of overall survival in EGFR-positive LAEEC patients. A nomogram has been developed, which demonstrates good performance in predicting patient outcomes. Notably, icotinib has proven to be beneficial for individuals in clinical stage III with favorable ECOG scores. These findings contribute to the development of more effective treatment strategies for EGFR-positive LAEEC patients, ultimately enhancing patient outcomes and overall survival rates.

## Data availability statement

The raw data supporting the conclusions of this article will be made available by the authors, without undue reservation.

## Ethics statement

The studies involving human participants were reviewed and approved by The Affiliated Huaian No. 1 People’s Hospital of Nanjing Medical University. Written informed consent for participation was not required for this study in accordance with the national legislation and the institutional requirements.

## Author contributions

Conceptualization, JW, HL and YS. Data curation, JW, JP, HL and YS. Formal analysis, JW, JP and YS. Investigation, YS. Methodology, JW, HL and YS. Resources, JW. Validation, YS. Visualization, YS. Writing – original draft, JW and JP. Writing – review & editing, JW, HL and YS. All authors contributed to the article and approved the submitted version.

## Supplementary material

Supplementary Figure 1 Immunohistochemical images of EGFR expression: **(A)** EGFR expression (+, low), **(B)** EGFR expression (++~+++, high).
