# Supplementary figures and images for: Development and internal validation of a nomogram for predicting survival of nonoperative EGFR-positive locally advanced elderly esophageal cancers
Source: Front Oncol. 2023 May 12;13:1097907. doi: 10.3389/fonc.2023.1097907 (PMC10213387; doi:10.3389/fonc.2023.1097907)

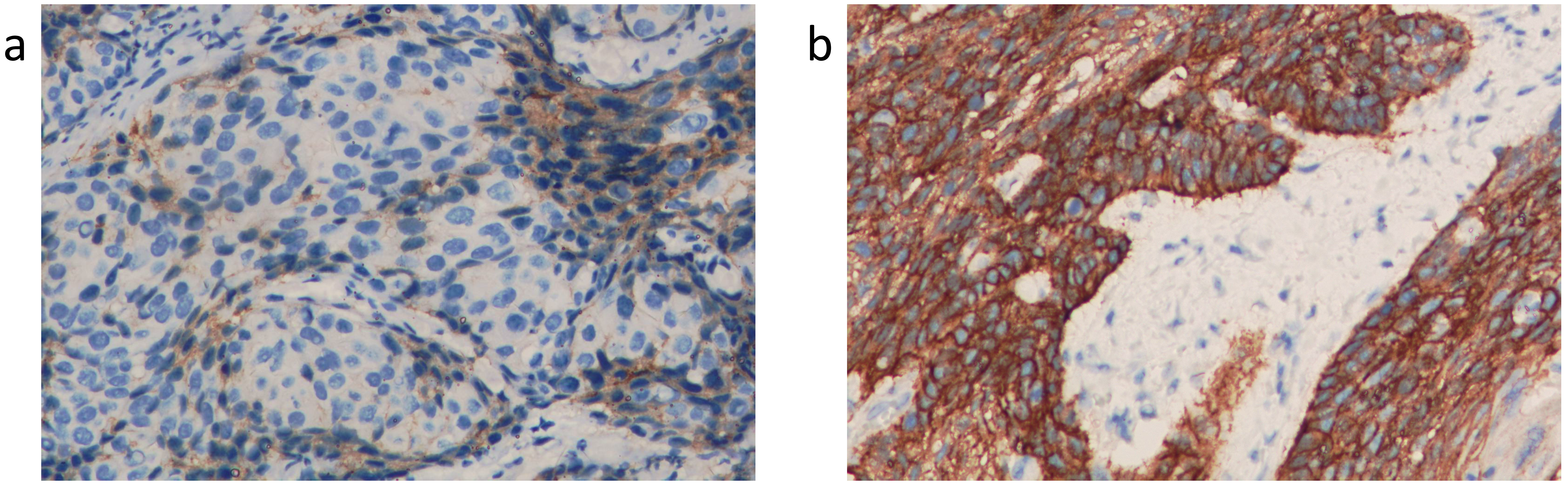

Supplement: Supplementary Figure 1 — Immunohistochemical images of EGFR expression: (A) EGFR expression (+, low), (B) EGFR expression (++~+++, high). [file Image_1.tif]
